# Prognostic factors and outcome of relapsed/progressive pediatric Ewing sarcoma: single-center 10-year experience

**DOI:** 10.1186/s43046-024-00232-4

**Published:** 2024-08-19

**Authors:** Omar Arafah, Reem Ragab Hegazy, Moatasem El Ayadi, Azza Mohamed Nasr, Mohamed Fawzy

**Affiliations:** 1 Department of Pediatric Oncology, National Cancer Institute, Cairo University, I Fom El Khaleeg Street, Kasr El Aini Avenue, Cairo, 11796 Egypt; 2 Department of Pediatric Oncology, Children’s Cancer Hospital Egypt 57357, Cairo, 11617 Egypt; 3 Department of Radiation Oncology, National Cancer Institute, Cairo University, Cairo, 11796 Egypt; 4 Department of Radiation Oncology, Children’s Cancer Hospital Egypt 57357, Cairo, 11617 Egypt

**Keywords:** Ewing sarcoma, Relapse-progression

## Abstract

**Background:**

Ewing sarcoma (ES) is the second most common primary malignant bone tumor in children and adolescents. Despite more intensive chemotherapy regimens and improved local control therapy, there is still a considerable rate of recurrent/progressive disease.

**Methods:**

A retrospective study of 50 relapsed/progressive ES patients who were treated at the National Cancer Institute (NCI), Cairo University, during the period from 1st of January 2008 to the end of December 2018, to assess different prognostic variables and disease outcomes.

**Results:**

Out of fifty eligible cases, 32 patients (64%) had disease recurrence, and 18 (36%) developed disease progression on treatment. The median follow-up period was 7.4 months. The median overall survival (OS) was 7.5 months, and the cumulative OS was 64% at 6 months and 32.6% at 1 year. The cumulative event-free survival (EFS) was 41.3% at 6 months and 22.3% at 1 year. Patients with disease recurrence had better OS and EFS than patients with disease progression (*p* = 0.019). Patients who underwent local control at relapse/progression had a significantly better outcome than patients who received chemotherapy only (*p* < 0.001). Recurrence > 2 years from initial diagnosis was the only independent predictor of better survival outcome.

**Conclusions:**

Patients with relapsing/progressive ES portended a poor outcome, with disease progression on treatment faring worse than relapse. Better outcome was observed in patients who experienced recurrence > 2 years after diagnosis, patients with disease recurrence rather than disease progression on treatment, and patients who underwent local control along with intensive chemotherapy.

## Introduction

Ewing sarcoma is the second most common high-grade sarcoma, arising in bone and soft tissue in children and adolescents and occurring mostly during the second decade of life [1]. Multiple treatment approaches, including local control with surgery, radiation, or a combination of both, along with systemic chemotherapy, improved the event-free survival to 69% and the overall survival to 72% in patients treated in the North American intergroup study INT-0091 [2].

Despite more intensive chemotherapy and improved local control measures during first-line treatment, the outcome for patients with metastatic disease at diagnosis remains poor, with relapse-free survival (RFS) ranging from 29% in lung exclusive metastasis to 8% in combined metastasis at initial presentation [3].

The prognosis for patients with refractory or recurrent Ewing sarcoma is always bad, with survival rates of 9.7% for event-free survival 5 years after a relapse and 13.8% for overall survival 5 years after a relapse [4].

Currently, there is no universally agreed second-line treatment for relapsed or refractory Ewing sarcoma [5]. Different chemotherapy regimens have shown responses; however, it is unknown whether one treatment is superior to the others [6]. Moreover, intensification to high-dose chemotherapy, total body irradiation, and autologous bone marrow transplantation did not improve the 2-year survival rate (20%) over conventional therapy [7].

In this study, we aimed to analyze the impact of different prognostic factors and salvage therapy on the outcome of patients with relapsed/progressive Ewing sarcoma.

## Patients and methods

This retrospective study included patients with relapsing/progressive Ewing sarcoma treated at the Pediatric Oncology Department, National Cancer Institute (NCI), Cairo University, Egypt, from January 1st, 2008, until the end of December 2018. Progression refers to patients who did not attain a complete or partial response during treatment, with the progression of target lesions while still on treatment. Relapse describes patients who re-developed the disease after going into complete remission after chemotherapy. Medical records were reviewed to extract demographic characteristics, date of presentation, initial investigations (imaging, pathological reports, bone marrow aspirate, and biopsy), and data of first-line treatment details (chemotherapy, radiotherapy, and surgery).

Data of relapse/progression were collected, including the timing of relapse/progression (during treatment, early within the first year of follow-up, or late after the first year), site (single or combined sites), whether detected during routine follow-up or suspected by clinical symptoms, and whether confirmed by biopsy or suggested by radiological findings only when surgical biopsies were not possible. Data on salvage therapy given at the time of relapse/progression were reviewed. Details of post-relapse/progression RT were collected (timing, dose, duration, and field). Response to first-line treatment or salvage therapy was assessed using RECIST criteria version 1.1 [8]. Survival outcome was evaluated at the median follow-up time.

### Treatment plan

#### First-line treatment protocol

All cases were initially treated according to the Children’s Oncology Group (COG) study (COGAEWS0031) [2, 9], consisting of cycles of vincristine, doxorubicin, and cyclophosphamide (VDC) alternating with cycles of ifosfamide and etoposide (I/E) for a total duration of 48 weeks. An interval-compressed regimen was implemented starting in 2017, where cycles alternated every 2 weeks instead of 3 for a total of 28 weeks, followed by rescue with colony-stimulating factors (CSF). Chemotherapy regimens were reviewed along with the local control modality used (surgery only, radiotherapy only, or surgery and radiotherapy).

### Treatment of relapse/progressive Ewing sarcoma

A Salvage chemotherapy (CTH)
  Variable combinations of chemotherapy were used as a second-line treatment:
    Regimen I: ICE (carboplatin IV 450 mg/m^2^ D1, etoposide IV 100 mg/m^2^ D1–D5, ifosfamide IV 1800 mg/m^2^ D1–D5) repeated every 21 days.
    Regimen II: VCR/TEM/IRN (vincristine IV 1.5 mg/m^2^ D1 and D8, temozolamide IV 100 mg/m^2^ D1–D5, irinotecan IV 20–40 mg/m^2^ D1–D5 and D8–D12) repeated every 21 days.
    Regimen III: docetaxel (TAX) + gemcitabine (GEM) (TAX IV 100 mg/m^2^ D8 and GEM IV 675 mg/m^2^ D1–D8) every 2 weeks.
B Radiation therapy (RT)
  Details of post-relapse/progression RT were collected (timing, dose, duration, and field). Local control at the time of relapse/progression either by definitive or post-surgical radiotherapy was done after re-evaluation, starting from the second to sixth cycle according to the response and primary management given. No lung re-irradiation was given during salvage therapy.
C Surgery
  Limb salvage, radical resection by amputation, or other surgical interventions, were done as salvage treatment for some cases after relapse/progression.
D Palliative treatment
  Palliative CTH was administered in the form of either oral etoposide (VP16) 50 mg/m^2^ for 21 days, with the cycles repeated every 28 days, or cyclophosphamide IV 750–1000 mg/m^2^ every 2–3 weeks. Palliative RT was given on the most painful sites in cases of nerve compression, along with pain killers.

### Statistical methods

Statistical analysis was done using IBM SPSS® Statistics version 22 (IBM® Corp., Armonk, NY, USA). Numerical data were expressed as mean and standard deviation, or median and range, as appropriate. Qualitative data were expressed as frequency and percentage. Pearson’s chi-square test or Fisher’s exact test was used to examine the relationship between qualitative variables. Survival analysis was done using Kaplan–Meier method, and a comparison between two survival curves was done using the log-rank test. Multivariate analysis was done using Cox-proportional hazard regression model for the significant factors affecting survival on univariate analysis. Hazard ratio (HR) with its 95% confidence interval (CI) was used for risk estimation. All tests were two-tailed. A *p*-value < 0.05 was considered significant. Post-relapse overall survival (PROS) was calculated from the date of the first relapse/progression to the date of death or the last follow-up. Post-relapse event-free survival (PREFS) was calculated from the date of the first relapse/progression to the date of further disease progression, second relapse, second malignancy, or death, whichever occurred first, or the date of the last follow-up.

## Results

During the study period, 153 patients presented with a confirmed diagnosis of the Ewing sarcoma family of tumors (ESFTs), 53 of whom (34.6%) had disease recurrence or progression. Three patients had missing initial data and were excluded from this study analysis. The remaining eligible cases (*n* = 50) included 32 patients with recurrent disease and 18 with disease progression. On enrollment, the median age was 14 years (range 1.5–26 years), with 43 cases (86%) being ≤ 18 years and 27 (54%) being females. Extremities were commonly affected (*n* = 31, 62%). Seventeen patients (34%) had metastatic disease, including 12 patients who had lung-only metastasis. Table 1 shows the demographic and clinical characteristics of the primary tumors.

**Table 1:** Demographic and clinical characteristics of included study patients (*n* = 50)

	**Value**
Age (years)	12.7 ± 5.2
Sex (male/female)	23/27
Primary tumor	
Axial skeleton	19 (38%)
Extremities	31 (62%)
The largest diameter > 8 cm	30 (60%)
Metastatic tumors	17 (34%)
Lung-only metastases	12 (24%)

Data are presented as mean ± SD or number (%)

### First-line management

**Fig. 1:**
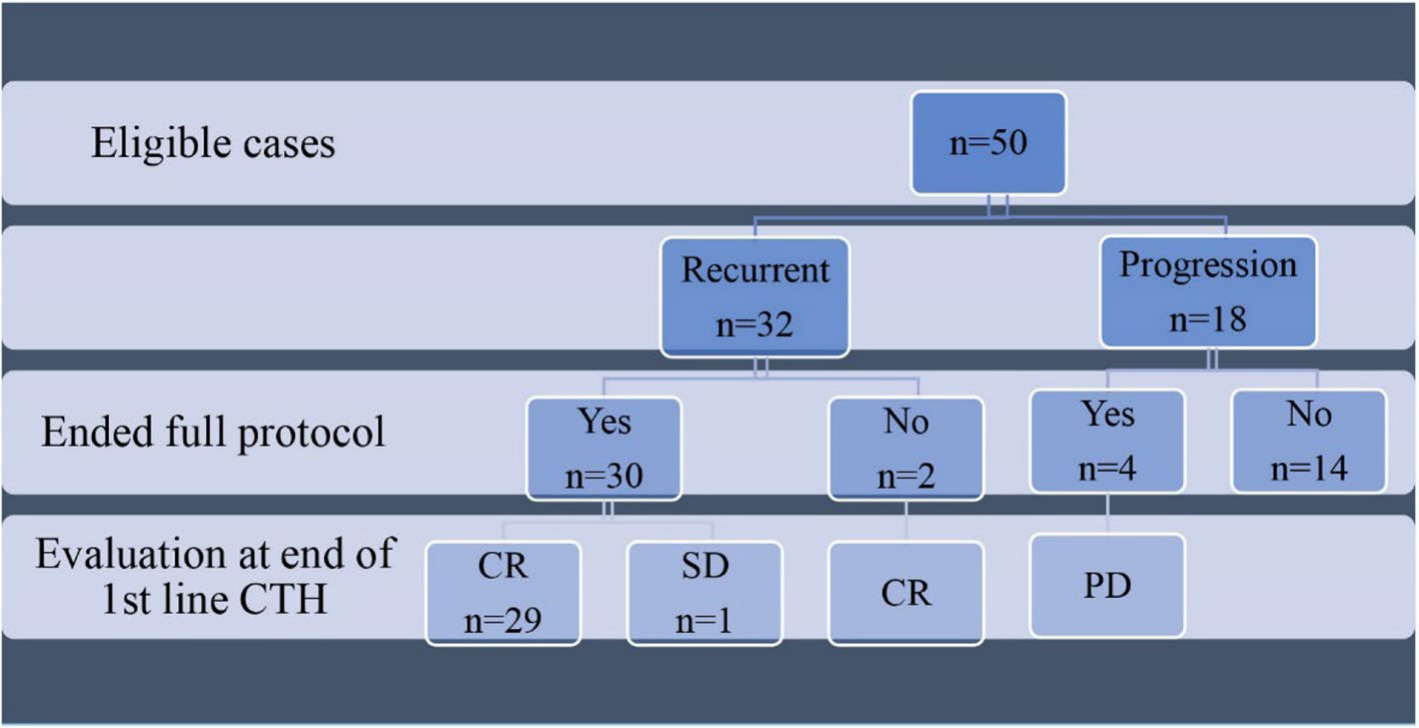
Number of recurrent/progressive patients and their response to 1st-line CTH

Table 2 shows the details of the first-line treatment of the primary tumors. All study patients were treated initially according to the COG-AEWS0031 protocol; 34 of them (68%) ended the full CTH protocol. Local control was achieved in 17 patients by surgery only (34%), in 14 patients by radiotherapy only (28%), or both (38%). Five patients (10%) received local radiotherapy plus a lung bath. Besides, 3 patients (6%) received a lung bath only. Therefore, the total number of patients who received radiotherapy was 36 (72%). According to RECIST criteria, 29 patients out of 34 who completed their CTH protocol (85%) achieved complete remission, one had a partial response, and four (12%) had progressive disease. Fourteen patients out of the 16 who did not complete the CTH protocol developed progression while on treatment, and two discontinued CTH (at weeks 39 and 42) due to severe adverse effects in the form of cardiac impairment. Both patients were in complete remission when they stopped CTH (Fig. 1).

**Table 2:** Initial disease management

		**Number (%)**
Initial chemotherapy with interval compression		9 (18%)
Ended full chemotherapy protocol		34 (68%)
Local control	Surgery	17 (34%)
	Radiotherapy	14 (28%)
	Both	19 (38%)
Tumor necrosis (*n* = 27)	≤ 90%	20 (74%)
	> 90%	7 (26%)
Surgical margin (*n* = 28)	Positive	1 (3.5%)
	Close	3 (10.7%)
Radiotherapy dose (Gy)	≥ 45	19 (60%)
	< 45	13 (40%)
Radiotherapy timing	At week 12	11 (35.5%)
	After week 12	20 (64.5%)
Radiotherapy duration	≤ 30 days	7 (22%)
	> 30 days	24 (78%)

### Disease characteristics at recurrence/progression

Recurrence or progression occurred within the first two years in 70% of cases. The site of the event is shown in Table 3. Half of the study patients developed only metastatic events, 10 of whom (40%) had an isolated pulmonary recurrence. Isolated local recurrences were found in 11 patients (22%) including 7 patients who did not receive local radiotherapy initially, while 14 patients (28%) had both local and metastatic recurrence/progression. Table 4 shows that the initial metastatic status was not associated with the pattern of failure at relapse/progression (*p* = 0.927).

**Table 3:** Clinical Characteristics at recurrence/progression

		**Number (%)**
Type of event	Recurrence	32 (64%)
	Progression	18 (36%)
Site of event	Local	11 (22%)
	Local and metastatic	14 (28%)
	Metastatic	25 (50%)
	Lung	10 (40%)
	Bone	10 (40%)
	Bone and lung	3 (12%)
	Bone and brain	2 (8%)
Time of event within 2 years		35 (70%)

**Table 4:** Relation between initial metastatic status and pattern of failure at relapse/progression

	**Site of relapse or progression**	**Number (%)**	***p*-value**
Initially localized disease (*n* = 33)	Local	8 (24.2%)	
	Local and metastatic	9 (27.3%)	
	Metastatic	16 (48.5%)	0.927
Initially metastatic disease (*n* = 17)	Local	3 (17.6%)	
	Local and metastatic	5 (29.4%)	
	Metastatic	9 (53%)	

**Fig. 2:**
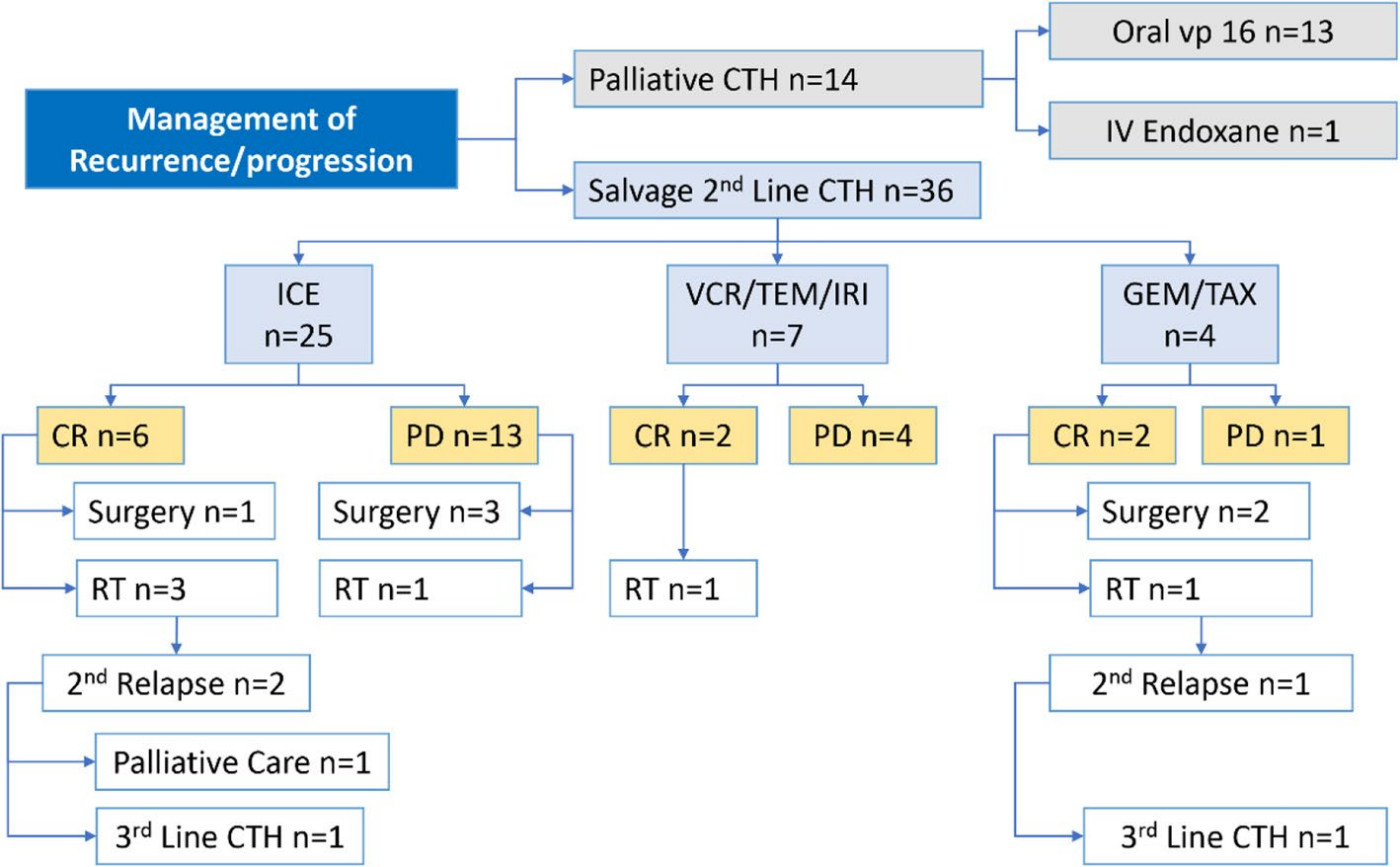
Treatment algorithm and disease response of study patients at recurrence/progression. VCR/TEM/IRN Vincristine/Temozolamide/Irinotecan, GEM/TAX Gemcitabine/Docetaxel, ICE Ifosfamide/Carboplatin/Etoposide, CTH chemotherapy, RT radiotherapy, CR complete remission, PD progressive disease

For management of recurrence/progression, 36 patients received salvage therapy, whereas the remaining 14 received palliative treatment (Fig. 2). Ten patients (27.7%) of those who received salvage therapy underwent local control by local radiotherapy (*n* = 4), definitive surgery (amputation or limb salvage, *n* = 4), or both surgery and RT (*n* = 2). Eighteen patients (50%) had tumor progression on salvage therapy; 10 patients went into a second remission, 4 died, and 4 discontinued treatment before the time of evaluation. Three out of 10 patients who went into second remission developed a second recurrence; one patient was referred to palliative care; and two received 3rd line CTH (ICE, VCR/TEM/IRN). Both patients had disease progression on the 3rd line CTH.

### Post-relapse survival analysis

The median follow-up period was 7.4 months (ranging from 1.12 to 58.13 months). During the period of the study, 43 patients died. The median overall survival (OS) was 7.56 months. The cumulative OS was 64% at 6 months and 32.6% at 1 year. The cumulative event-free survival (EFS) at 6 months and 1 year were 41.3% and 22.3%, respectively.

### Effect of initial presentation and initial management

**Fig. 3:**
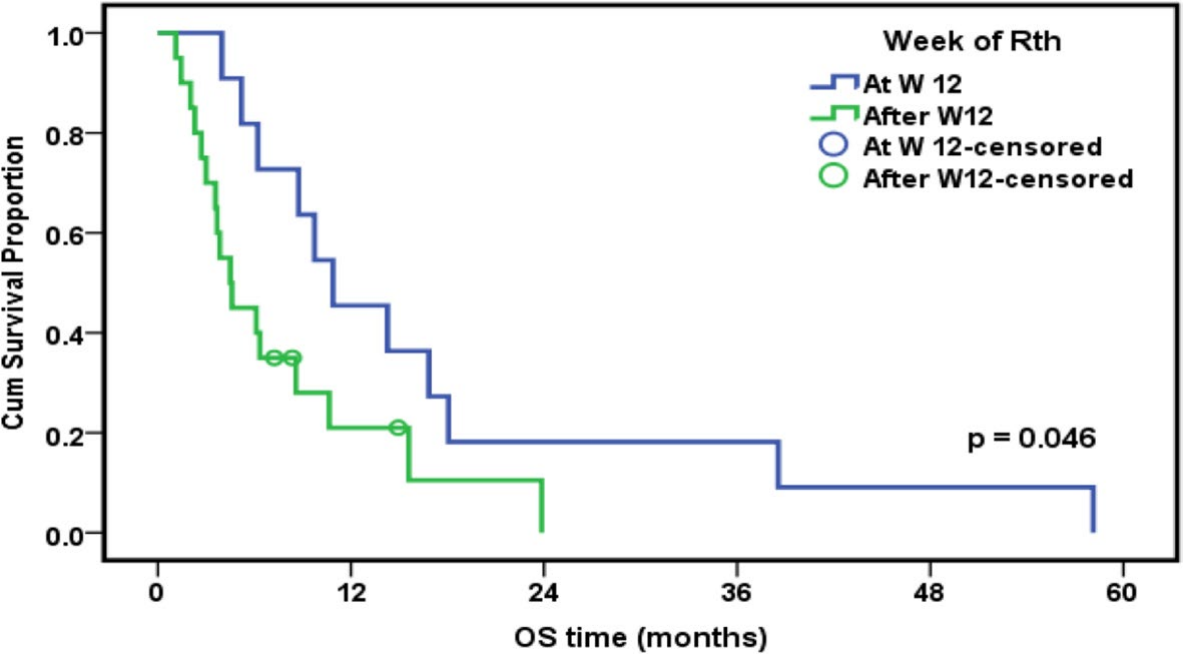
Overall survival and timing of RTH in patients with recurrent/progressive ES

**Fig. 4:**
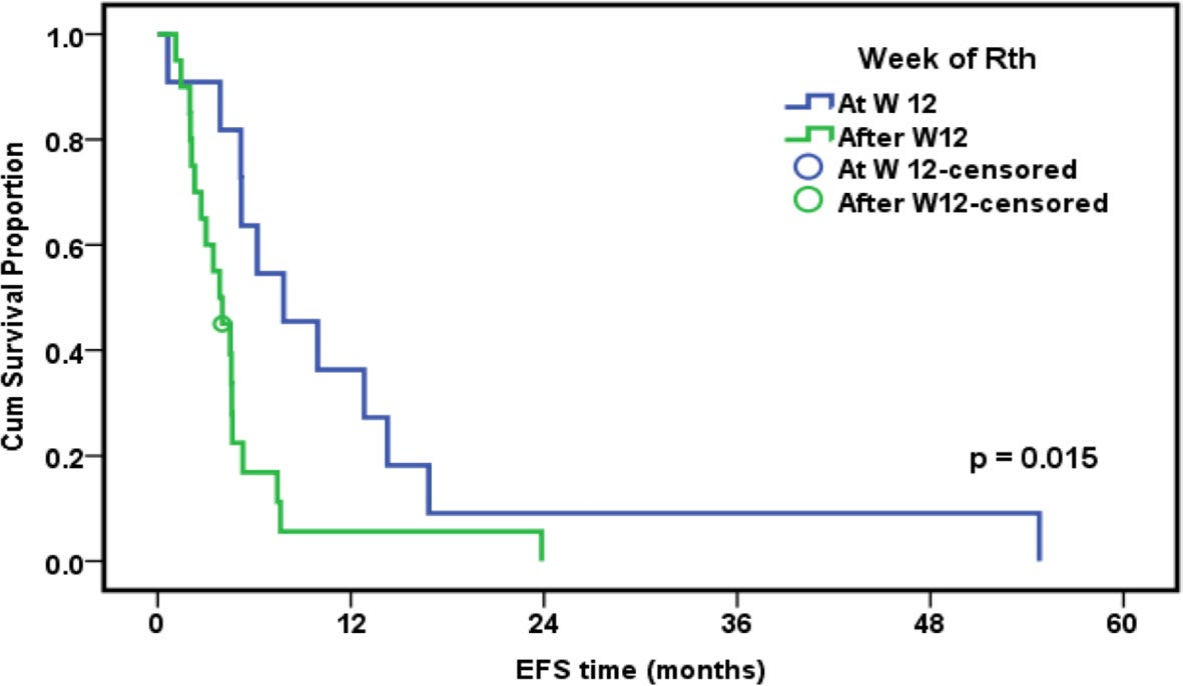
Event-free survival and timing of RTH in patients with recurrent/ progressive ES

There was no effect of the initial patient’s demographic and clinical characteristics on EFS and OS at 6 months. Patients who had local control by surgery plus RT had an inferior survival outcome than patients who had surgery or RT alone for local control (Table 5). Likewise, survival outcome was significantly affected by the timing of RT, with better OS and EFS at 6 months for those receiving RT at week 12 (*p* = 0.046 and 0.015, respectively) (Figs. 3 and 4).

**Table 5:** Survival outcome of study patients in relation to the demographic and initial disease characteristics

	**No**	**No. of events**	**Cumulative EFS (%) at 6 months**	***P*-value**	**No. of deaths**	**Cumulative OS (%) at 6 months**	***P*-value**
Whole group	50	46	41.3		43	64	
Age				0.242			0.653
≤ 18 years	43	39	46		37	62.8	
> 18 years	7	7	14.3		6	57.1	
Gender				0.754			0.878
Male	23	21	41.7		19	69.6	
Female	27	25	40.7		24	59.3	
Initial site				0.117			0.272
Axial skeleton	19	17	45.1		16	63.2	
Extremity	31	29	35.5		27	64.5	
Initially metastatic				0.191			0.238
Metastatic	17	16			15	52.9	
Not metastasis	33	30	45.5		28	69.7	
Interval compression				0.715			0.571
Yes	9	7	27.8		5	66.7	
No	41	39	44		38	63	
Local control				0.036			0.053
Surgery	17	14	57		13	64.3	
Radiotherapy	14	13	47		12	71.4	
Both	19	19	21		18	47.4	
Timing of radiotherapy^a^				0.015			0.046
At w12	11	11	54.5		11	72.7	
Post w12	20	19	16.9		17	40	

^a^Data about timing of radiotherapy were available for 31 patients out of 33 total patients who received radiotherapy

### Effect of recurrence/progression and its management

**Fig. 5:**
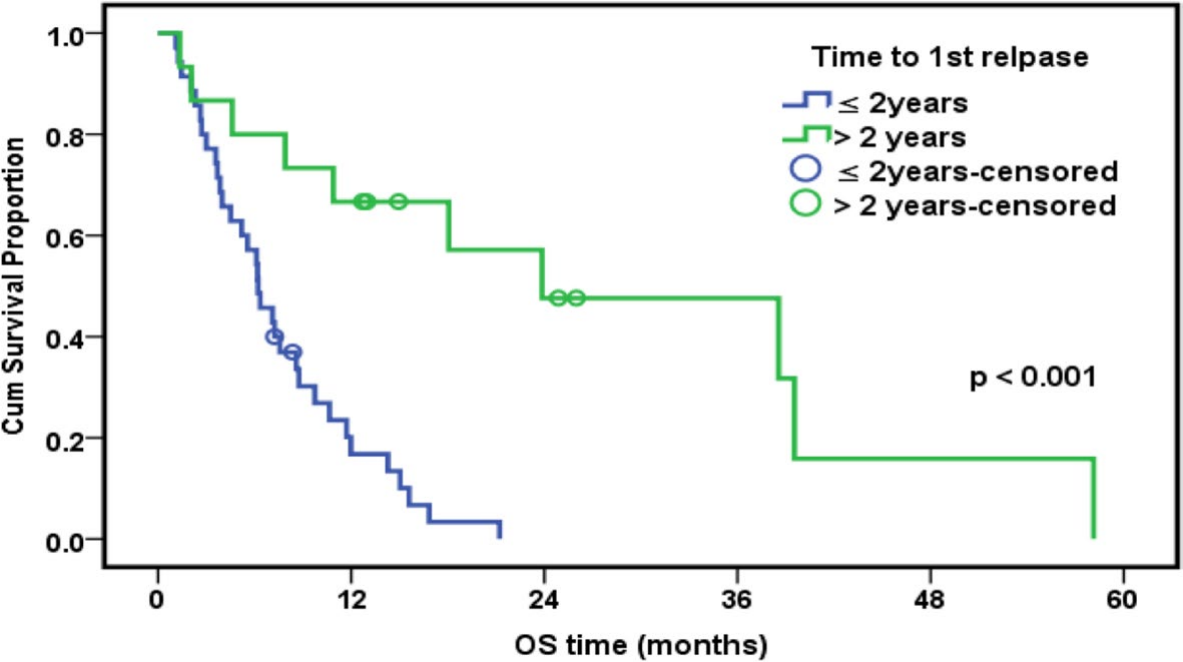
Overall survival and time to first event in patients with recurrent/progressive ES

**Fig. 6:**
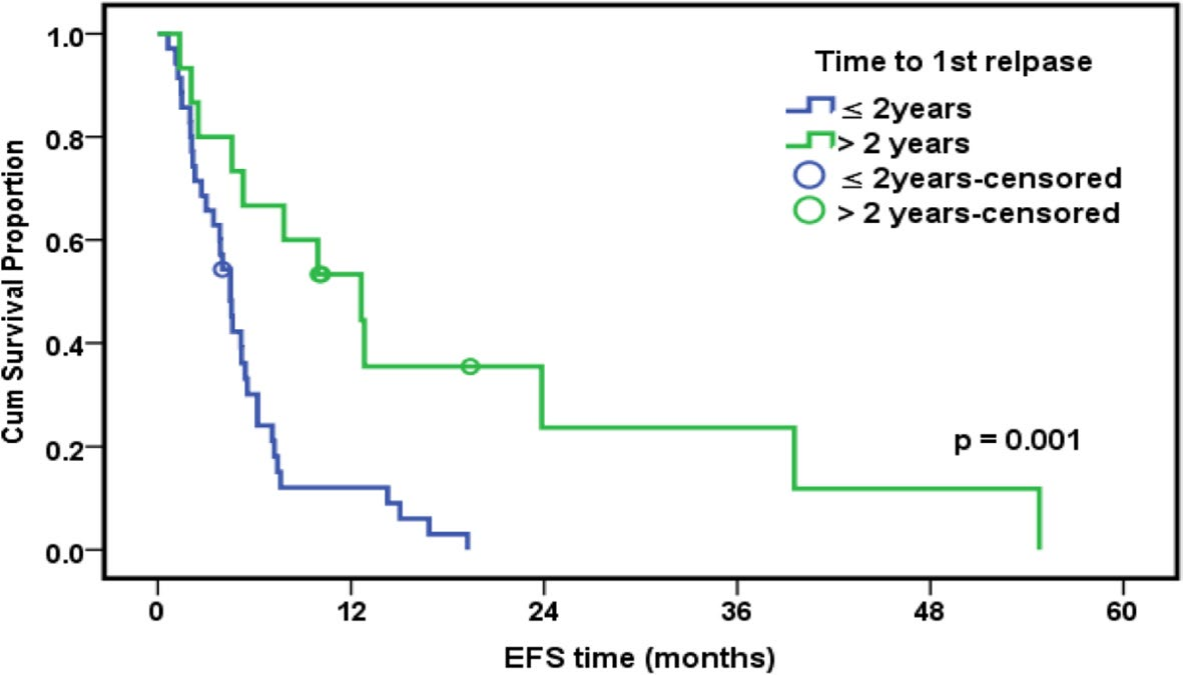
Event-free survival and time to first event in patients with recurrent/progressive ES

**Fig. 7:**
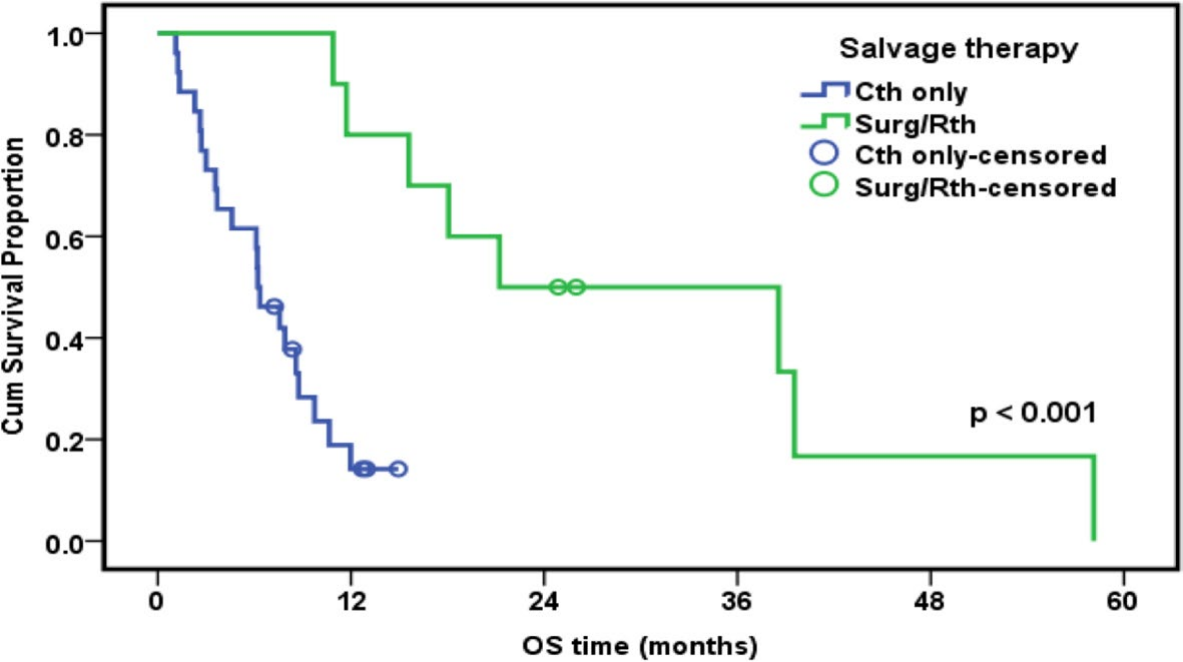
Overall survival and salvage therapy given in patients with recurrent/progressive ES

**Fig. 8:**
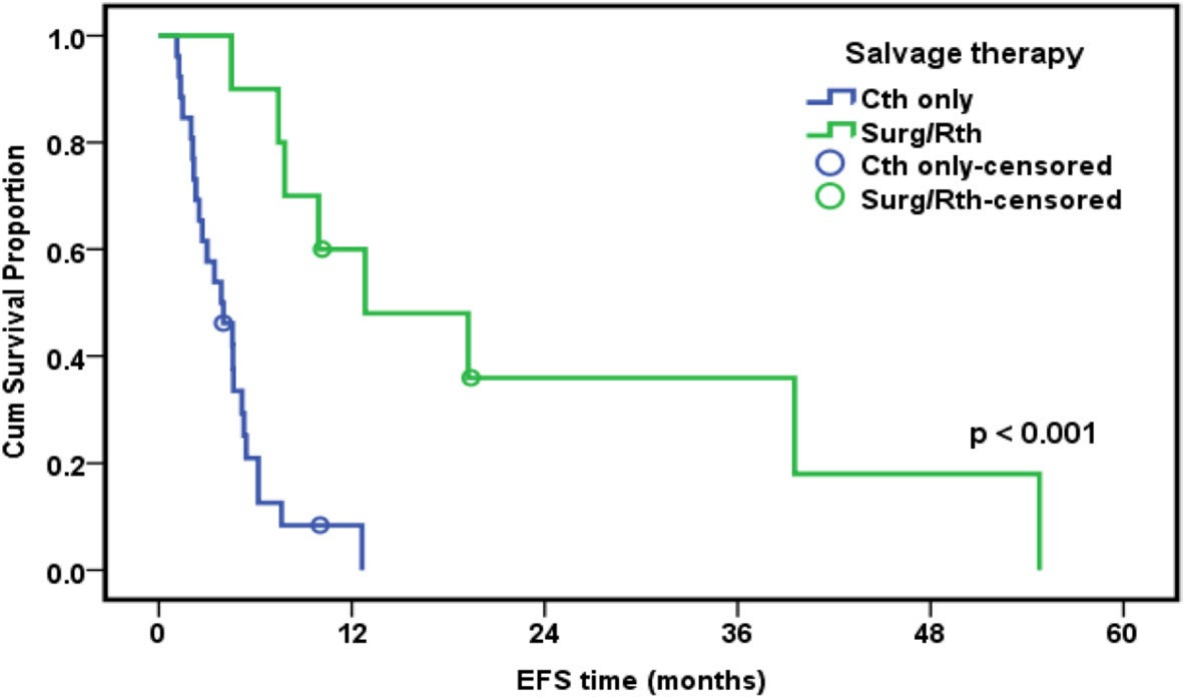
Event-free survival and salvage therapy given in patients with recurrent/progressive ES

Early recurrence or progression (within 2 years of the initial diagnosis) had a significantly worse OS and EFS at 6 months than those with later events (*p* < 0.001 and *p* = 0.001, respectively) (Figs. 5 and 6). OS at 6 months was significantly better in patients who had disease recurrence than those who developed disease progression while on treatment (*p* = 0.019). Similarly, disease recurrence was associated with better EFS, with a near-significant difference (*p* = 0.059). The site of the event had a near-significant effect on OS at 6 months, where local recurrence/progression was associated with better OS than metastatic or local and metastatic events (*p* = 0.052). Patients who underwent local control (surgery, RT, or both) had better survival outcomes than patients who received salvage CTH only (*p* < 0.001 for both OS and EFS) at 6 months (Table 6, Figs. 7 and 8). Metastatic relapses tend to occur earlier (≤ 2 years) than local relapses in study patients. In multivariate analysis, the time to first recurrence was the only independent factor predicting OS (*p* < 0.001, HR, 5.4 (95% CI, 2.1–13.6). Similarly, the time to first recurrence was the only independent factor predicting EFS (*p* = 0.003, HR, 3.2 (95% CI, 1.5–6.9).

**Table 6:** Survival outcome of study patients in relation to the recurrence/progression status and management

	***n***	***n* events**	**Cumulative EFS (%) at 6 months**	***P*-value**	***n* deaths**	**Cumulative OS (%) at 6 months**	***P*-value**
Type of event				0.059			0.019
Recurrence	32	30	50		27	68.8	
Progression	18	16	25		16	50.0	
Site of event				0.137			0.052
Local	11	9	54.5		8	81.8	
Metastatic	25	23	34.9		21	52.0	
Local and metastatic	14	14	35		14	57.0	
Site of metastasis				0.2			0.11
Isolated pulmonary	10	9	12.5		7	60	
Others	15	14	40		14	40	
Time to 1st event				0.001			< 0.001
≤ 2 years	35	34	30.2		33	57.1	
> 2 years	15	12	66.7		10	37.7	
Salvage therapy				< 0.001			< 0.001
CTH only	26	24	21		21	58.0	
CTH + surgery ± RT	10	8	80		8	90.0	
Type of salvage CTH				0.892			0.164
ICE	25	23	40		21	64	
Other CTH	11	9	32.7		8	72.7	

*RT* radiotherapy, *CTH* chemotherapy

Hematology toxicity was observed in 4 patients (8%), and one or more episodes of febrile neutropenia were recorded in 15 (30%). Five patients died of sepsis. Cardiac toxicity in the form of heart failure during first-line CTH was reported in 2 patients (4%). No pulmonary toxicity was recorded.

## Discussion

Although more patients with newly diagnosed Ewing sarcoma get into remission with modern multidisciplinary treatment approaches and are potential long-term survivors, relapse remains a considerable clinical obstacle against maintaining a favorable frontline therapy response. About 25% of patients with an initially localized disease will relapse after first-line treatment, and the recurrence rate is even higher in an initially metastatic disease [10].

Several prognostic factors have been identified, which can help in establishing future therapy plans. Several salvage chemotherapy regimens have been used with variable responses in patients with recurrent Ewing sarcoma, but the superiority of one regimen over another has not yet been proven, and toxicities in those previously heavily treated patients should be considered [6]. It has not yet been established what the optimal number of chemotherapy cycles is for relapsed patients, which depends in part on response, tolerance of therapy, toxicities, and quality of life. Likewise, despite the local control that is usually offered, it is mostly non-feasible, especially for widespread metastatic recurrences [6].

In the present retrospective study of 50 patients with recurrent/progressive ES, we identified some risk factors that have effects on survival outcomes after disease recurrence/progression. Only 20% of patients who had disease recurrence achieved a second remission that was maintained in almost half of them with a median OS of 7.5 months. The age at presentation, gender, and initial disease site did not affect survival outcome. In concordance with these findings, previous studies did not demonstrate an effect of the initial disease site on survival outcome [11, 12].

Primary local control in Ewing sarcoma is achieved through surgery and/or radiotherapy. In the present study, patients who had their primary local control by both surgery and RT had an inferior outcome compared to patients who had local control by either surgery or RT alone. According to Becker et al., a significantly better EFS was associated with local control by surgery alone (71.7%) followed by postoperative RT (64.1%) rather than definitive RT alone (30%) (*p* = 0.009). While in tune with our study, the cumulative incidence of local and distant failure in the Becker et al. study at 2 and 5 years was 25% for surgery alone, 11% for radiotherapy alone, and 16.7% for surgery plus radiotherapy, but this did not reach statistical significance (*p* = 0.64) [11]. This might be explained in part by the fact that patients who had local control by both modalities (surgery and RT) in our study were those patients whose tumors showed poor response to chemotherapy [TN: tumor necrosis (< 90%)] and/or surgical margins positive for tumor infiltration, and might also be due to interrupted courses of RT.

A COG study conducted by Womer et al. [13] on a large number of Ewing sarcoma patients tested whether chemotherapy intensification through interval compression could improve the outcome. They concluded that for localized Ewing sarcoma, chemotherapy administered every 2 weeks is more effective than chemotherapy administered every 3 weeks, with no increase in toxicity. Interval-compressed therapy had no apparent effect on local recurrence alone but decreased the incidence of distant and combined relapses. The 5-year OS and EFS were significantly better in every 2-week regimen [13]. Our study did not show a significant effect of interval compression of first-line CTH on survival outcome, possibly due to the small number of cases, yet it should be noted that the majority of our cases (*n* = 41) received their chemotherapy every 3 weeks.

Patients with combined local and distant relapses have the worst outcomes, while those with isolated local recurrences appear to do better, as shown in our study and other studies [4]. This is understandable given that these patients may have a lower total burden of disease at recurrence and may be amenable to further local therapies in addition to systemic treatment.

More debatable is the prognostic impact of isolated pulmonary recurrence, as some series have reported that these patients do better than those with other distant site metastases. Stahl et al. [14] and Bacci et al. [4] found a better prognosis for patients diagnosed with pulmonary metastases only at the time of relapse compared to extrapulmonary lesions. Other studies did not find such an association with survival improvement [15], in agreement with the current study. However, the small number of patients with isolated pulmonary recurrence in our study (*n* = 10) precluded a definitive conclusion.

In multivariate analysis, the time to first recurrence was the only independent predictor of post-recurrence survival outcome. Many studies showed that most recurrences present within 2 years after primary diagnosis, while rarely occurring after the fifth year [4, 14]. In our study, 15 patients (30%) who had a late recurrence (> 2 years from the initial diagnosis) had a better survival outcome compared with 35 (70%) who had recurrences within 2 years. This is consistent with other reports that also confirm the importance of time to recurrence as a prognostic factor affecting the survival outcome of recurrent ES patients [4, 12, 15–18]. Our study showed that local recurrences occur with higher frequency after 2 years, which may explain in part the favorable prognosis of later recurrences.

A variety of chemotherapy regimens have produced responses in patients with recurrent Ewing sarcoma, with no evident superiority of one over another [6]. The retrospective nature of our study and the small number of patients did not permit an extensive analysis of each salvage regimen separately. However, since the ICE combination reportedly is an effective salvage treatment for patients with recurrent ES [6, 19] and is the most commonly used regimen in our study, we analyzed the impact of this regimen on outcomes versus other regimens used. There was no significant relation between the type of salvage CTH (ICE vs. others) and survival outcome. On the other hand, aggressive local measures along with intensive chemotherapy improved the outcomes compared to chemotherapy alone.

Overall, the prognosis of recurrent Ewing sarcoma is constantly dismal, and the outcome remains poor. The standard approach to their management has not yet been established, and many patients may initially benefit from salvage chemotherapy in terms of reducing symptoms and prolonging time to further progression, but consistent cures remain elusive. Knowledge of prognostic factors that affect the survival of these patients may help guide future therapy decisions.

In conclusion, our study showed that the main factors predictive of improved post-relapse survival in relapsing/progressive ES were prolonged time to first recurrence, local site of recurrence rather than metastatic or combined, relapse rather than progression and salvage CTH combined with surgery and/or RT, compared to CTH alone. The time to the first recurrence was the only independent factor predicting post-relapse survival. There was no prognostic significance for sites of metastasis (isolated pulmonary or other sites), type of salvage CTH (ICE or other regimen), or metastatic status at initial presentation.

## Limitations of the study

This study was conducted retrospectively with no control of patient assignment to different variables, compliance to treatment, or completeness of data collected. The relatively small number of study patients precluded proper statistical inference for many studied variables.

## Declarations

Ewing sarcoma is the second most common high-grade sarcoma, arising in bone and soft tissue in children and adolescents. Despite improved event-free survival of up to 70% achieved with multidisciplinary approaches, recurrence and progression during treatment remain a real nightmare to the clinicians involved in the management of this disease. Limited options that could be offered to those already heavily treated patients urged us to review our management experience in the Pediatric Oncology Department at the National Cancer Institute, Cairo University, Egypt, over around 10 years, trying to find out possible adverse factors that may be involved in scaling the cure rate, hoping to modify some changeable variables in our practice. Moreover, adding to the existing knowledge helps in reaching definitive conclusions more clearly in debatable areas of management. Later recurrences of more than 2 years and local rather than metastatic and combined recurrences were associated with the best outcome.

## Data Availability

The datasets used and/or analyzed during the current study are available from the corresponding author on reasonable request.
